# CRISPR/Cas9-mediated genome editing identifies six testis-expressed genes individually dispensable for overt male fecundity in mice

**DOI:** 10.7555/JBR.39.20250247

**Published:** 2026-07-28

**Authors:** Heling Fu, Haoran Chen, Chenmeijie Li, Shuai Lu, Yayun Gu, Zhibin Hu

**Affiliations:** 1 State Key Laboratory of Reproductive Medicine and Offspring Health, Nanjing Medical University, Nanjing, Jiangsu 211166, China; 2 Department of Epidemiology, Center for Global Health, School of Public Health, Nanjing Medical University, Nanjing, Jiangsu 211166, China

**Keywords:** spermatogenesis, genetics of male infertility, dispensable genes, CRISPR/Cas9

## Abstract

The genetic landscape of male infertility is highly complex. It is estimated that at least 1000–2000 genes are involved in human and mouse infertility. Although functional analyses have been performed on hundreds of genes, many others still have unknown functions. Generating gene-editing mice is a powerful tool for exploring whether a given gene is essential for male reproduction *in vivo*. In the current study, we investigated the function of six genes, *Efcab7*, *Tekt3*, *Mlf1*, *Rp1l1*, *Agbl2*, and *Tmsb15a*, using the clustered regularly interspaced short palindromic repeats (CRISPR)/CRISPR-associated protein 9 (Cas9) system. Individual disruption of these six genes did not cause overt male sterility in mice under standard breeding conditions. Phenotypic analyses at 8–10 weeks of age generally showed that most reproductive parameters were comparable to those of wild-type controls; however, we observed significant sperm motility alterations in *Tekt3*-mutant mice (decreased motility) and *Rp1l1*-mutant mice (increased motility), as well as flagellar abnormalities in *Tekt3* mutants. In summary, these findings suggest that complete or near-complete disruption of the tested murine alleles does not cause overt male infertility, providing useful functional evidence for interpretation of candidate variants while not excluding human-specific or allele-specific effects.

## Introduction

Male infertility is a global health concern, affecting more than 50 million couples worldwide according to the World Health Organization (WHO)^[1]^. Genetic disorders are a major cause of male infertility^[2]^. Next-generation sequencing technologies have identified key genes crucial for spermatogenesis, including *SPEM2*^[3]^, autophagy-related *ATG5/ATG7*^[4]^, and *FBXO47*^[5]^. Previously, we have also identified several genes, such as *WDR63*^[6]^ and *CCER1*^[7]^. However, with more than 1000–2000 genes predominantly expressed in the testis^[8]^, the genes currently established as causative account for only a small proportion of the genetic basis of male infertility.

Humans and mice share a high degree of genetic similarity, with approximately 99% of mouse genes having a human counterpart^[9]^. This genetic homology provides a strong foundation for using mice as models to study human diseases. Many genes have already been shown to decrease fertility in mouse models upon loss of function, indicating their indispensability for male reproduction. Meanwhile, hundreds of genes have also been identified as non-essential for male fertility, likely due to functional redundancy. For example, studies have reported more than 20 genes, such as *Allc*^[10]^, *Fscb*^[10]^, *Tmem210*^[11]^, and *4921539E11Rik*^[11]^, that are dispensable for male fertility. Notably, patients with male infertility often seek assistance from *in vitro* fertilization or intracytoplasmic sperm injection to have their own offspring. However, there is a significant risk that pathogenic genes or variants may be transmitted to their progeny during these processes if preimplantation genetic diagnosis is not performed^[12–14]^. Therefore, screening the functions of as many testis-expressed genes as possible in organismal models is crucial. This approach helps identify not only genes that are dispensable for male fertility but also those critical for sperm formation and/or function.

In the current study, we generated six mouse lines (*i.e.*, *Efcab7*, *Tekt3*, *Mlf1*, *Rp1l1*, *Agbl2*, and *Tmsb15a*) deficient in testis-expressed genes using the clustered regularly interspaced short palindromic repeats (CRISPR)/CRISPR-associated protein 9 (Cas9) system. Among these genes, five (*Efcab7*, *Tekt3*, *Mlf1*, *Rp1l1*, and *Agbl2*) were identified as carrying variants in whole-exome sequencing data from tetralogy of Fallot (TOF) cases and have been reported to be associated with cilia-related functions^[15–20]^; *Tmsb15a* was selected as a cancer-testis gene with testis-enriched expression, which is hypothesized to play a potential role in spermatogenesis^[21]^. Considering the structural similarities between cilia and sperm flagella, and the potential importance of testis-enriched genes in reproductive development for the latter, we conducted comprehensive reproductive phenotypic analyses of these models. Our results demonstrate that individual disruption of these six genes did not cause overt male sterility in mice under standard breeding conditions. These findings could help avoid unnecessary and duplicative research efforts by other investigators.

## Materials and methods

### Animals

All animal experiments performed in the current study were approved by the Institutional Animal Care and Use Committee (IACUC) of Nanjing Medical University (Nanjing, China) in compliance with guidelines and regulations for animal experiments (Approval No. IACUC-1912049). All six mouse lines were generated using CRISPR/Cas9 techniques.

### Generation of gene-edited mice using the CRISPR/Cas9 system

In this study, we generated six mouse lines using the CRISPR/Cas9 system. Five of these genes (*Efcab7*, *Tekt3*, *Mlf1*, *Rp1l1*, and *Agbl2*) were identified as harboring variants in TOF cases and were selected based on their conserved roles in ciliary/flagellar function. To recapitulate these clinical variants, we aimed to generate knock-in (KI) mouse models; due to technical constraints, we successfully established one KI line, while for the remaining four TOF candidates, we performed targeted knockout (KO) models designed at or near the variant sites. Additionally, *Tmsb15a* was selected as a representative cancer-testis gene with testis-enriched expression, for which a KO model was generated to evaluate its potential role in spermatogenesis. The Cas9 plasmid (Addgene, Watertown, MA, USA) was linearized and transcribed into mRNA *in vitro* using a T7 Transcription Kit (Ambion, Austin, TX, USA). The single guide RNAs (sgRNAs) used in the current study are listed in ***Supplementary Table 1***. Cas9 mRNA and sgRNA were microinjected into the fertilized superovulated wild-type C57BL/6 female mouse zygotes, which had been mated with wild-type C57BL/6 male mice. After injection, the zygotes were transferred into the oviducts of pseudopregnant ICR females. Founder mice were screened by PCR and Sanger sequencing to confirm the mutations. Subsequently, these founder mice were backcrossed to the C57BL/6 background for at least three generations to ensure a pure genetic background. Homozygous and heterozygous mutant mice were obtained through sibling crosses of the backcrossed offspring.

Genomic DNA was collected from tail biopsies, and the mutation of the target gene was confirmed by PCR and Sanger sequencing. The primers and PCR conditions for genotyping are listed in ***Supplementary Table 2***.

### RNA extraction, reverse transcription PCR (RT-PCR) and reverse transcription-quantitative PCR (RT-qPCR)

Total RNA was extracted and purified from multiple adult tissues using the TRIzol reagent (Vazyme, Nanjing, China) according to the manufacturer's instructions, and was reverse transcribed into cDNA using the HiScript Ⅱ Q RT SuperMix (Vazyme). All tissues, including the heart, liver, spleen, lung, kidney, brain, muscle, testis, colon, and stomach, were collected from 8–10-week-old C57BL/6J mice. RT-PCR was performed using 2× Phanta Max Master Mix (Dye Plus) (Vazyme), and the products were separated by electrophoresis on an agarose gel. RT-qPCR was carried out using ChamQ Universal SYBR qPCR Master Mix (Vazyme) on a QuantStudio™ 7 Flex Real-Time PCR System (Thermo Fisher Scientific, Waltham, MA, USA). Relative mRNA levels were normalized to the endogenous *Gapdh* gene (internal control). The primers used for PCR are listed in ***Supplementary Table 3***.

### Phylogenetic tree analysis

Phylogenetic trees (method: UPGMA) for each gene were constructed using DNAMAN software (version 7.212, Lynnon Corp., Quebec, Canada) based on alignments of the amino acid sequences from *Homo sapiens, Mus musculus,* and other mammal species. The sequences were downloaded from the NCBI database.

### Fertility test

Sexually mature C57BL/6 wild-type or gene-edited male mice aged 8–10 weeks were housed individually with two adult wild-type female mice for at least two months. Male mice were removed after a vaginal plug was detected, and females were kept for another three weeks to count the final litters. To evaluate fertility, each male mouse was treated as the experimental unit, and the litter size was averaged per male before comparison. The number of males, total pups, recorded litters, recorded litters per male, and male-level mean litter size (presented as mean ± standard deviation [SD]) were recorded and are summarized in ***Table 1***, whereas latency to pregnancy was not systematically tracked.

**Table 1:** Male fertility of six mouse lines

Gene symbols	Genotype	No. of males	Total pups (*n*)	Recorded litters	Recorded litters per male	Male-level mean litter size (mean ± SD)	*P* value
Wild type	+/+	4	105	12	3, 3, 3, 3	8.75 ± 0.74	–
*Efcab7*	c.509-1G>C/c.509-1G>C	3	98	12	4, 4, 4	8.17 ± 0.63	0.343
*Tekt3*	−76bp/−76bp	3	66	8	3, 3, 2	8.33 ± 0.58	0.543
*Mlf1*	R243*/R243*	4	150	16	5, 3, 4, 4	9.50 ± 1.00	0.257
*Rp1l1*	−7bp/−7bp	3	114	14	4, 4, 6	8.08 ± 0.52	0.257
*Agbl2*	−11bp/−11bp	3	96	12	4, 4, 4	8.00 ± 0.25	0.200
*Tmsb15a*	−191bp, G>A/Y	3	98	12	4, 4, 4	8.17 ± 0.95	0.400
*P* values were calculated using two-sided exact permutation tests comparing the mean litter size per male between each mutant line and the wild-type group, with individual male mice used as the unit of analysis.Abbreviation: SD, standard deviation.							

### Morphological and histological analysis of the testis and epididymis

The testes and epididymides were extracted from at least three wild-type and gene-edited male mice aged 8–10 weeks following euthanasia. After photographing and measuring the body and testicular weights, the testes and epididymides were fixed in modified Davidson's fluid for 48 h. Subsequently, the organs and tissues were dehydrated, embedded in paraffin, and cut into 5-μm-thick sections. After deparaffinization with xylene, the sections of the testes and epididymides were stained with periodic acid-Schiff (PAS) and Mayer's hematoxylin and eosin (HE), respectively. The sections were then imaged and observed using an Olympus BX microscope equipped with an Olympus DP20 color camera (Olympus, Tokyo, Japan).

### Computer-assisted sperm analysis (CASA)

Sperm from each mutant mouse aged 8–10 weeks and age-matched wild-type C57BL/6 controls were collected from the cauda epididymis, suspended in human tubal fluid (HTF) culture medium (InVitroCare, Frederick, MD, USA), and maintained at 37 ℃. The sperm were then diluted, placed on an 80-μm chamber slide, and analyzed using the IVOS Ⅱ analyzer (Hamilton Thorne, Beverly, MA, USA), except for *Tmsb15a*. The sperm parameters of the *Tmsb15a*^−/Y^ line and its corresponding WT controls were measured using the TOX IVOS Ⅱ analyzer (Hamilton Thorne), which may introduce systematic deviations when comparing these data with those obtained from the other five lines. Nevertheless, both WT and *Tmsb15a*^−/Y^ mice were assayed on the same system; therefore, comparisons between these two groups are valid. The proportion of motile spermatozoa and the spermatozoa concentration (M/mL) were measured and analyzed.

### Morphological analysis of spermatozoa

Three male mice aged 8–10 weeks from each gene-edited line and age-matched wild-type C57BL/6 controls were used for malformation analysis. Cauda epididymal spermatozoa were suspended in human tubal fluid culture medium (InVitroCare), and 10 μL of epididymal sperm suspension fluid was placed and spread out on a poly-L-lysine (PLL)-coated glass slide. The slides were air-dried and then immersed in 4% paraformaldehyde solution for 1 h. The slides were stained with HE solution prior to imaging and observed using an Olympus BX microscope equipped with an Olympus DP20 color camera (Olympus). At least 200 sperm on each slide were counted, and abnormal sperm were labeled using ImageJ software.

### Protein extraction and Western blotting analysis

Mouse testicular tissues were collected from wild-type or gene-edited mice aged 8–10 weeks after removal of the tunica albuginea. The samples were lysed in RIPA buffer (Beyotime, Shanghai, China) supplemented with a protease inhibitor cocktail (MedChemExpress, Monmouth Junction, NJ, USA), followed by ultrasonication and incubation on ice for 30 min. After centrifugation at 4 ℃, the supernatant was collected and mixed with 2× SDS loading buffer (Applygen, Beijing, China). Proteins were denatured by heating at 100 ℃ for 10 min. The denatured proteins were then separated on an SDS-polyacrylamide gel and transferred to a polyvinylidene fluoride (PVDF) membrane (Millipore, Billerica, MA, USA) for immunoblot analysis. The antibodies used in the current study were: mouse anti-MLF1 (1∶250, Cat. #sc-514294, Santa Cruz Biotechnology, Santa Cruz, CA, USA), rabbit anti-GAPDH (1∶1000, Cat. #2118L, Cell Signaling Technology, Danvers, MA, USA), anti-mouse IgG HRP (1∶2000, Cat. #A0216, Beyotime), and anti-rabbit IgG HRP (1∶2000, Cat. #A0208, Beyotime).

### Statistical analysis

Continuous phenotypic variables were compared using two-tailed unpaired Student's *t*-tests. For fertility analyses, individual male mice were treated as the experimental units, and male-level mean litter sizes were compared between each mutant line and wild-type group using two-sided exact permutation tests. A two-sided *P* value < 0.05 was considered statistically significant. All data are represented as the mean ± standard deviation (SD) from at least three replicates for each experiment. Microsoft Excel and GraphPad Prism 9 were used for statistical analysis.

## Results

### Expression patterns and conservation analyses of candidate genes

**Figure 1:**
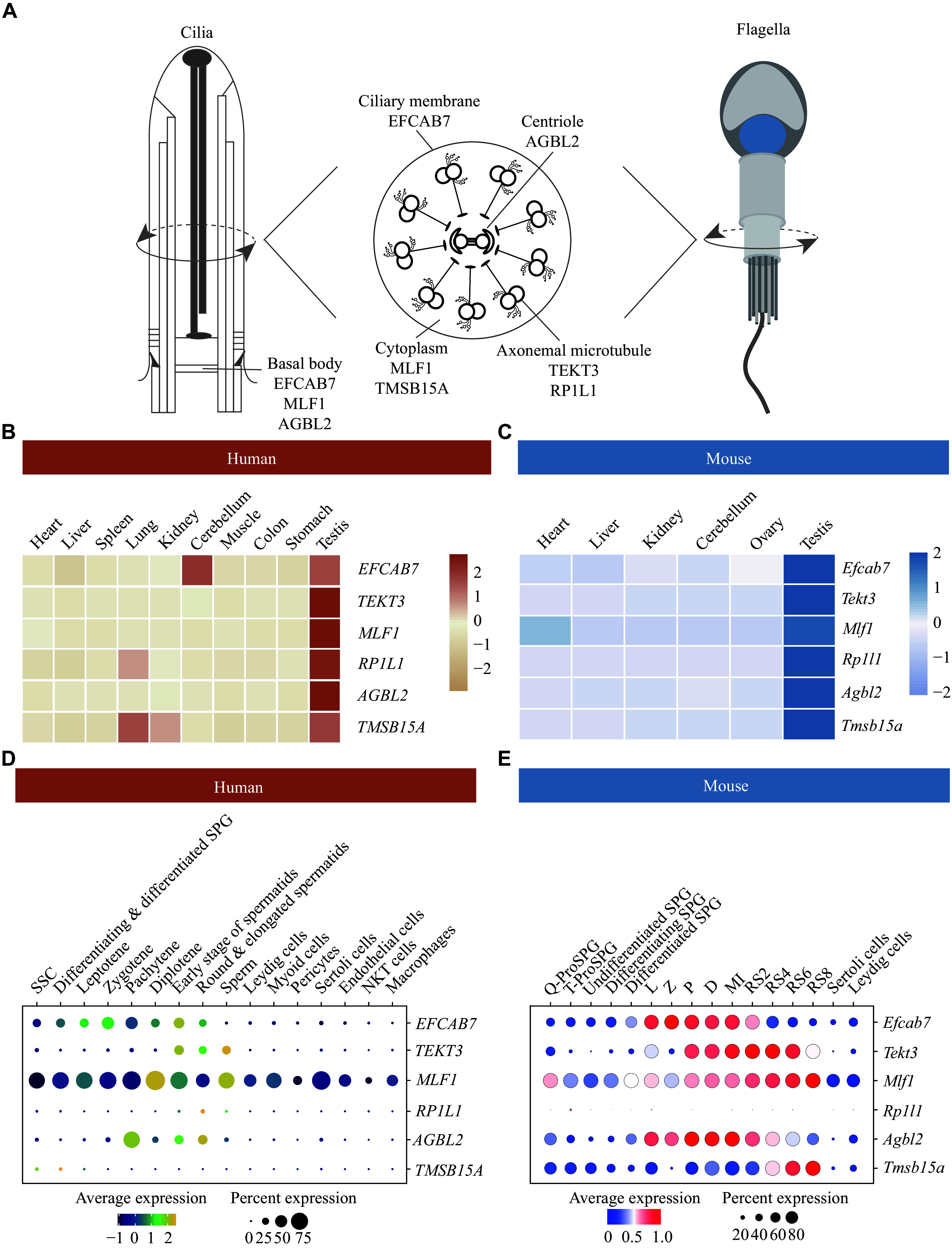
Subcellular localization and expression patterns of specific genes in humans and mice. A: Schematic diagram of cilia and sperm flagella with the subcellular localization of six genes depicted. B: Expression of the six genes in different human tissues. Data sourced from the GTEx public database. C: Expression of the six genes in different mouse tissues. Data sourced from published literature^[24]^. D: Single-cell expression of the six genes during human spermatogenesis. E: Single-cell expression of the six genes during mouse spermatogenesis. Abbreviations: L, leptotene; D, diplotene; MⅠ, metaphase Ⅰ; P, pachytene; Q-ProSPG, quiescent progenitor spermatogonia; RS2–RS8, round spermatids at steps 2–8; SPG, spermatogonia; SSC, spermatogonial stem cell; T-ProSPG, transit-amplifying progenitor spermatogonia; Z, zygotene.

Cilia and sperm flagella both share the "9 + 2" structure (***Fig. 1A***), and any alteration in their structure or function can lead to serious diseases, such as primary ciliary dyskinesia^[22–23]^ and male infertility^[6]^. Here, we explored the function of six genes with different subcellular localizations in cilia (***Fig. 1A***). Among these genes, *EFCAB7* is situated within the ciliary membrane^[15]^, while *MLF1*^[18]^ and *TMSB15A*^[15]^ are found in the cytoplasm; *AGBL2*^[20]^ is localized to the centriole and, along with *MLF1*^[18]^ and *EFCAB7*^[16]^, is also localized to the basal body. Additionally, *TEKT3*^[17]^ and *RP1L1*^[19]^ are associated with axonemal microtubules. To further investigate the function of these six genes in male fertility using mouse models, we first ensured that the target genes met two criteria: (i) high expression in the testis (testis-expressed) to minimize potential side effects in other systems and (ii) the presence of a mouse ortholog for functional validation. As shown in ***Fig. 1B*** and ***1C***, all six genes showed substantial or relatively high expression in the testes of both humans and mice, although their tissue-specific expression patterns differed. To confirm this, we extracted RNA from multiple mouse tissues, including the heart, liver, spleen, testis, *etc.*, and performed conventional RT-PCR and RT-qPCR. The results consistently showed a high expression level in the testis for all six genes (***Supplementary Fig. 1A***). *Rp1l1* also exhibited considerable expression in other murine tissues, such as the lung, in addition to its highest expression in the testis. Using single-cell RNA sequencing (scRNA-seq) data generated from human and mouse spermatogenic cells at different stages, we found that these six genes exhibited varying expression levels throughout spermatogenesis (***Fig. 1D*** and ***1E***). *Efcab7* and *Agbl2* showed elevated expression in early spermatids or pachytene in both humans and mice; *Tekt3*, *Mlf1*, and *Tmsb15a* showed varying expression biases during different stages of spermatogenesis in humans and mice, suggesting a degree of heterogeneity between species. Although *Rp1l1* showed high expression at the tissue level, scRNA-seq data showed very low expression levels in all cells during spermatogenesis, including those in the microenvironment.

Moreover, to understand the evolutionary conservation of these candidate genes among mammals, we constructed phylogenetic trees and found that all six proteins showed evolutionary conservation across the examined mammalian species (***Supplementary Fig. 1B***).

### Generation of mutant mouse lines

To investigate the essentiality of six candidate target genes in spermatogenesis, we generated six mutant mouse lines using the CRISPR/Cas9 system. The mutant alleles were detected by genomic PCR and confirmed by Sanger sequencing. The mutated loci of all mutant mouse lines are summarized in ***Supplementary Table 1***. The primers used for genotyping and their annealing temperatures are shown in ***Supplementary Table 2***.

### Fertility tests of mutant male mice

To examine the fecundity of these mutant males, individual mutant male mice aged 8–10 weeks were caged with up to two wild-type female mice for at least two months. The fecundity of four wild-type males was tested in parallel as positive controls. Litter sizes of all mutant lines were comparable to those of wild-type controls, and no significant differences were detected (***Table 1***, ***Supplementary Table 4***). Thus, no overt reduction in fecundity was detected for these mutant lines under standard laboratory breeding conditions.

### Phenotypic analyses of mutant males

Although all mutant males had normal fertility, we conducted detailed analyses of reproductive phenotypes, including testicular and sperm development, for each mutant line.

**Figure 2:**
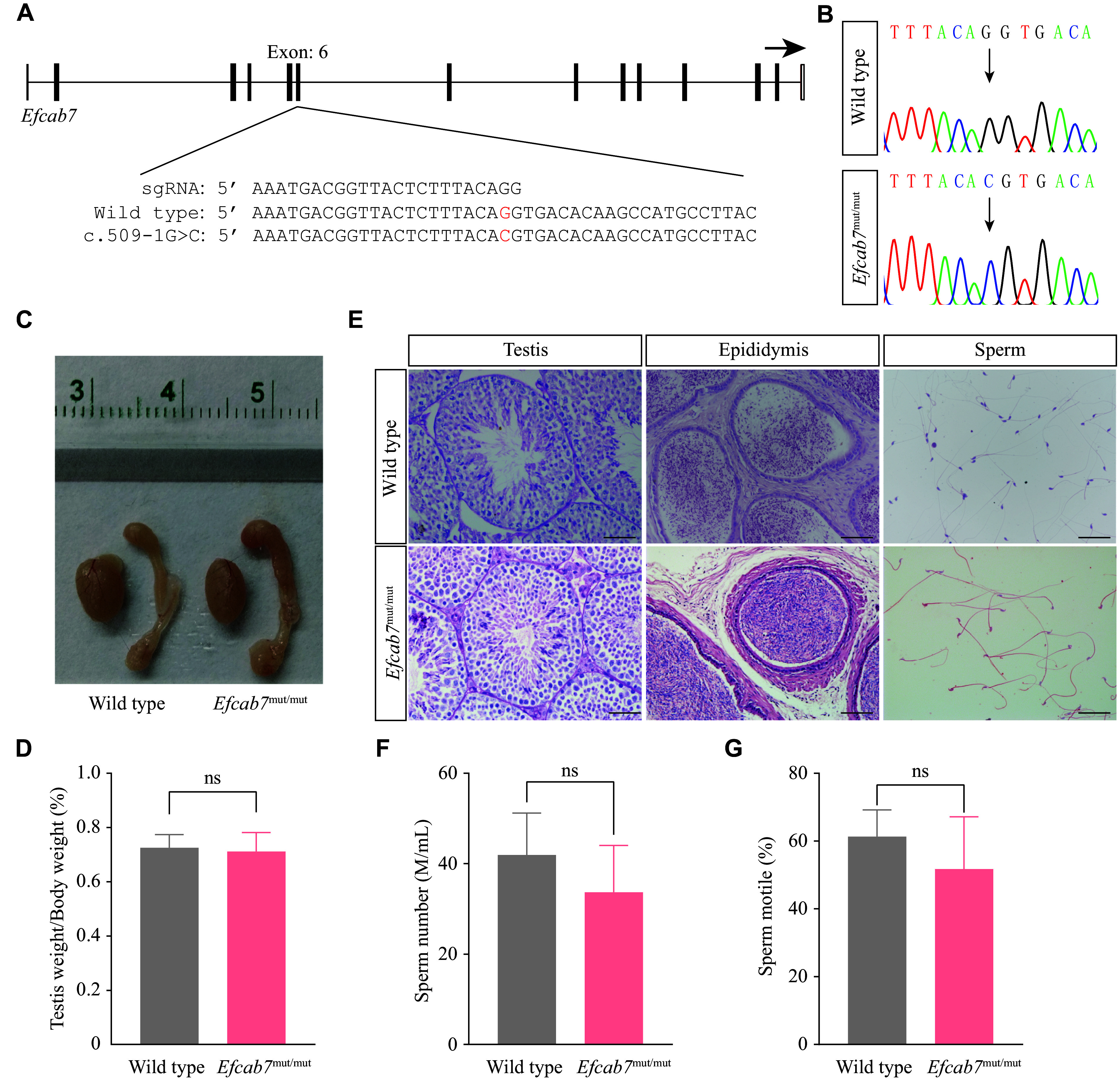
Phenotypic analysis of *Efcab7*^mut/mut^ male mice. A: Gene editing strategy for *Efcab7*. An sgRNA was designed to target upstream of exon 6 in intron 5. B: Genotyping of *Efcab7*^mut/mut^ mice through PCR and subsequent Sanger sequencing. Arrows indicate the mutant alleles. C: Morphology of the testes from wild-type and *Efcab7*^mut/mut^ mice. A cm-scale ruler is shown above for size reference. D: Comparison of bilateral testis weight to body weight between wild-type mice and *Efcab7*^mut/mut^ mice. *n* = 3 males per group. E: Histological analysis of the testes, epididymides, and sperm in wild-type and *Efcab7*^mut/mut^ mice. Testis sections were stained with PAS; epididymal sections and sperm smears were stained with HE. Scale bars, 50 μm (testes), 100 μm (epididymides), and 50 μm (sperm). F: Comparison of sperm concentration between wild-type and *Efcab7*^mut/mut^ mice. *n* = 3 males per group. M/mL, million per milliliter. G: Comparison of sperm motility between wild-type and *Efcab7*^mut/mut^ mice. *n* = 3 males per group. Quantitative data of D, F, and G are presented as mean ± standard deviation (SD), and statistical analysis was performed using two-tailed unpaired Student's *t*-test. Abbreviation: ns, not significant.

We previously reported that *Efcab7* mutant mice exhibited hindered ciliary transport and disrupted cardiac development^[25]^. Briefly, we generated *Efcab7* mutant lines by designing an sgRNA targeting the region upstream of exon 6 within intron 5 of the *Efcab7* gene, resulting in a G>C transversion at the splicing site. These changes were confirmed by genomic PCR and Sanger sequencing (***Fig. 2A*** and ***2B***), mimicking the variant detected in one TOF case (***Supplementary Table 5***). The primers used for genotyping are summarized in ***Supplementary Table 2***. In addition to cardiac phenotypes, we also examined their reproductive phenotypes. No statistically significant difference was observed in testis size and weight between the wild-type and homozygous knock-in mice (***Fig. 2C*** and ***2D***). Similar to control samples, histological sections of knock-in testes and epididymides showed normal spermatogenesis in the seminiferous tubules and normal sperm morphology and density in the cauda epididymides (***Fig. 2E***). Moreover, CASA results revealed that *Efcab7*^mut/mut^ spermatozoa exhibited normal concentration and motility compared with control spermatozoa (***Fig. 2F*** and ***2G***).

**Figure 3:**
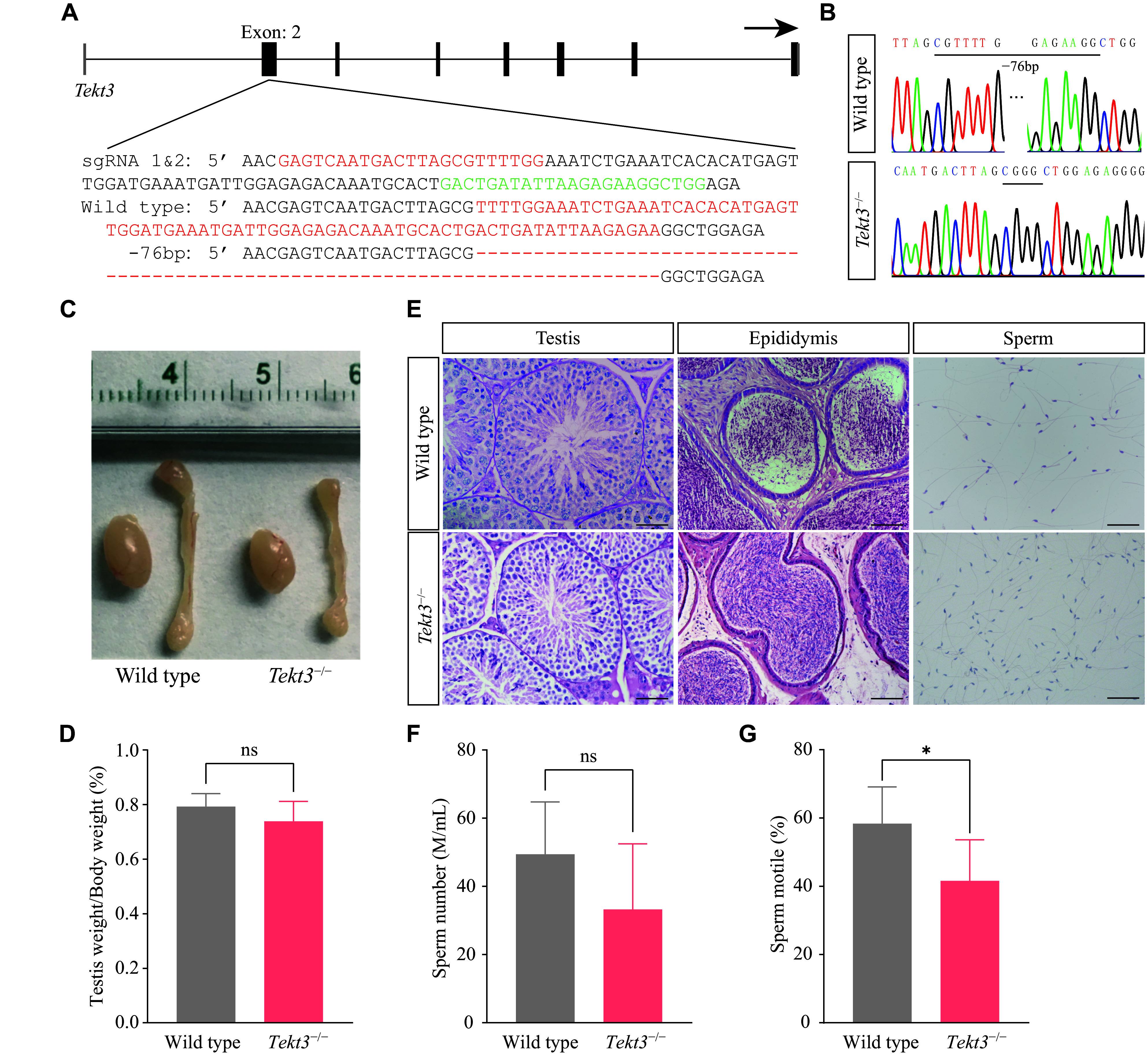
Phenotypic analysis of *Tekt3*^−/− ^male mice. A: Gene editing strategy for *Tekt3*. Two sgRNAs were designed to target exon 2 of *Tekt3*. B: Genotyping of *Tekt3*^−/−^ mice through PCR and subsequent Sanger sequencing. Lines indicate the mutant alleles. C: Morphology of testes from wild-type and *Tekt3*^−/−^ mice. A cm-scale ruler is shown above for size reference. D: Comparison of bilateral testis weight to body weight between wild-type mice and *Tekt3*^−/−^ mice. *n* = 3 males per group. E: Histological analysis of the testes, epididymides, and sperm in wild-type and *Tekt3*^−/−^ mice. Testis sections were stained with PAS; epididymal sections and sperm smears were stained with HE. Scale bars, 50 μm (testes), 100 μm (epididymides), and 50 μm (sperm). F: Comparison of sperm concentration between wild-type and *Tekt3*^−/−^ mice. *n* = 3 males per group. M/mL, million per milliliter. G: Comparison of sperm motility between wild-type and *Tekt3*^−/−^ mice. *n* = 3 males per group. Quantitative data of D, F, and G are presented as mean ± standard deviation (SD), and statistical analysis was performed using two-tailed unpaired Student's *t*-test. Abbreviation: ns, not significant.

*Tekt3* is a gene containing eight exons. To disrupt this gene, we designed two sgRNAs targeting exon 2 (***Fig. 3A***), resulting in a 76-bp deletion of exon 2 (***Fig. 3B***). The primers used for identifying the mutant allele are presented in ***Supplementary Table 2***. The *Tekt3* mutant mice were viable, and no statistically significant difference was observed in testis size and weight between the homozygous mutant and wild-type mice (***Fig. 3C*** and ***3D***). Morphological and histological analyses of *Tekt3*^−/−^ testes and epididymides were then performed. The results showed that histological sections of mutant testes and epididymides exhibited normal morphology and structure compared with wild-type controls (***Fig. 3E***). CASA also revealed that *Tekt3*^−/−^ spermatozoa exhibited normal concentration compared with control spermatozoa (***Fig. 3F***). We also observed that the motility of *Tekt3*^−/−^ spermatozoa was significantly lower than that of wild-type spermatozoa; however, the motility of *Tekt3*^−/−^ spermatozoa exceeded 40%, the progressive motility was comparable with wild-type (***Supplementary Fig. 2***), and *Tekt3*^−/−^ male mice were fertile. To investigate the underlying cause of reduced sperm motility in *Tekt3*^−/−^ male mice, we conducted comprehensive morphological analyses of over 300 sperm from both wild-type and *Tekt3*^−/−^ mice separately. The results showed no significant differences in head morphology between the two groups, but *Tekt3*^−/−^ males frequently exhibited prominent flagellar bending, with an incidence of 9.99% compared with 3.25% in wild-type mice (***Supplementary Table 6***). This increased frequency of flagellar abnormalities may contribute to the reduced sperm motility observed in *Tekt3*^−/−^ mice.

**Figure 4:**
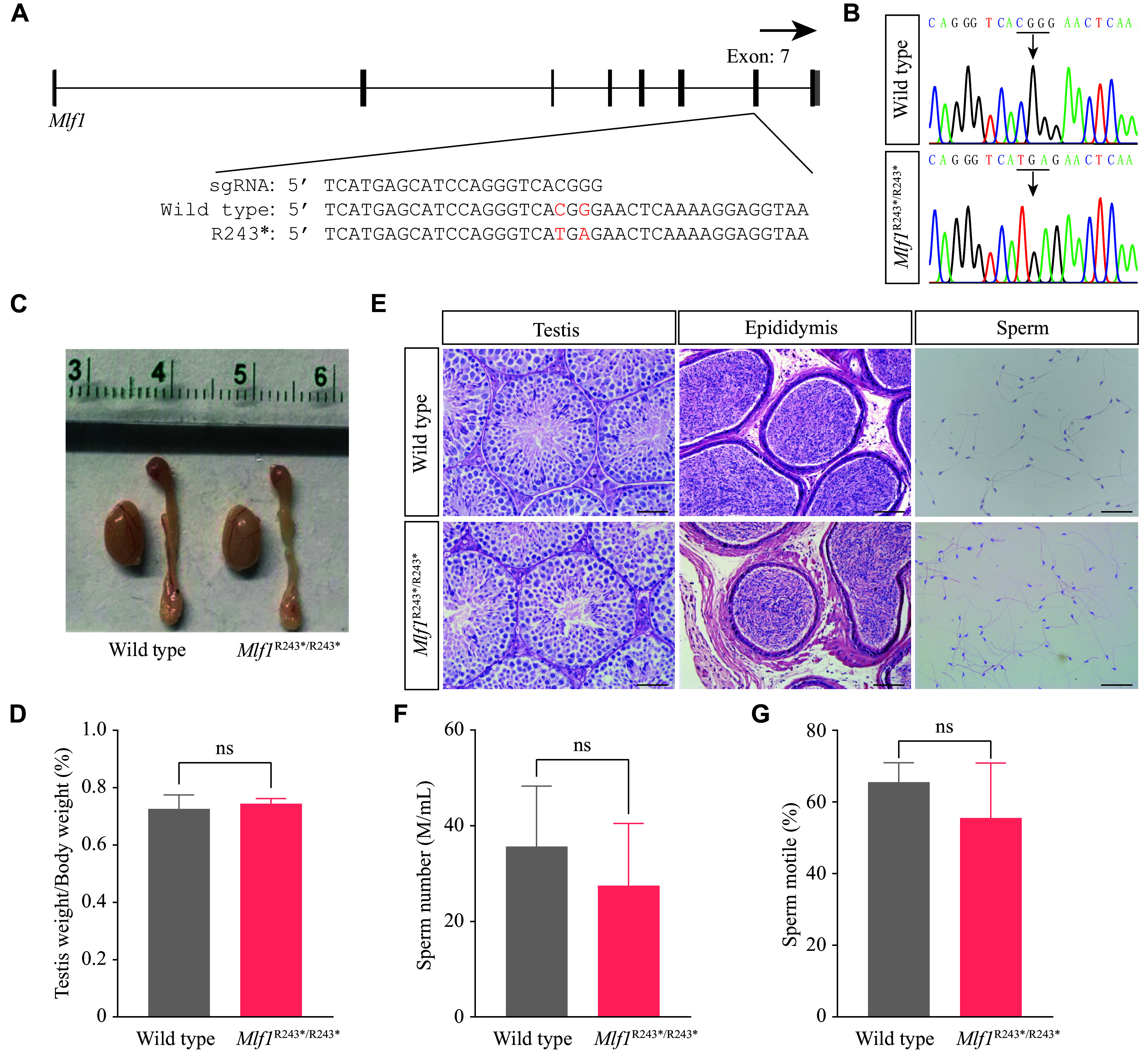
Phenotypic analysis of *Mlf1*^R243*/R243*^ male mice. A: Gene editing strategy for *Mlf1*. An sgRNA was designed to target exon 7 of *Mlf1*. B: Genotyping of *Mlf1*^R243*/R243*^ mice through PCR and subsequent Sanger sequencing. Arrows indicate the mutant alleles. C: Morphology of the testes from wild-type and *Mlf1*^R243*/R243*^ mice. A cm-scale ruler is shown above for size reference. D: Comparison of bilateral testis weight to body weight between wild-type mice and *Mlf1*^R243*/R243*^ mice. *n* = 3 males per group. E: Histological analysis of the testes, epididymides, and sperm in wild-type and *Mlf1*^R243*/R243*^ mice. Testis sections were stained with PAS; epididymal sections and sperm smears were stained with HE. Scale bars, 50 μm (testes), 100 μm (epididymides), and 50 μm (sperm). F: Comparison of sperm concentration between wild-type and *Mlf1*^R243*/R243*^ mice. *n* = 3 males per group. M/mL, million per milliliter. G: Comparison of sperm motility between wild-type and *Mlf1*^R243*/R243*^ mice. *n* = 3 males per group. Quantitative data of D, F, and G are presented as mean ± standard deviation (SD), and statistical analysis was performed using two-tailed unpaired Student's *t*-test. Abbreviation: ns, not significant.

*Mlf1* is located in the cytoplasm and the cilium basal body. To disrupt *Mlf1*, we designed an sgRNA targeting its exon 7 to mimic the nonsense variant detected in one TOF case (***Fig. 4A*** and ***Supplementary Table 5***). Then, we performed PCR using the primers presented in ***Supplementary Table 2*** to identify the mutant allele. Mutant mice carrying a CGG>TGA transition (R243*) were confirmed by PCR and Sanger sequencing (***Fig. 4B***). RT-qPCR analysis demonstrated that the mutation led to a significant downregulation of *Mlf1* mRNA expression (***Supplementary Fig. 3A***), which was accompanied by decreased protein abundance (***Supplementary Fig. 3B***). The observed protein reduction was moderate, likely due to the mutation's distal position within the coding region. We found that the testis size, weight, and histology of *Mlf1*^R243*/R243*^ males were comparable to those of control mice (***Fig. 4C***–***4E***). Additionally, there were no significant differences between wild-type and mutant males in sperm concentration and the percentage of motile spermatozoa (***Fig. 4F*** and ***4G***).

**Figure 5:**
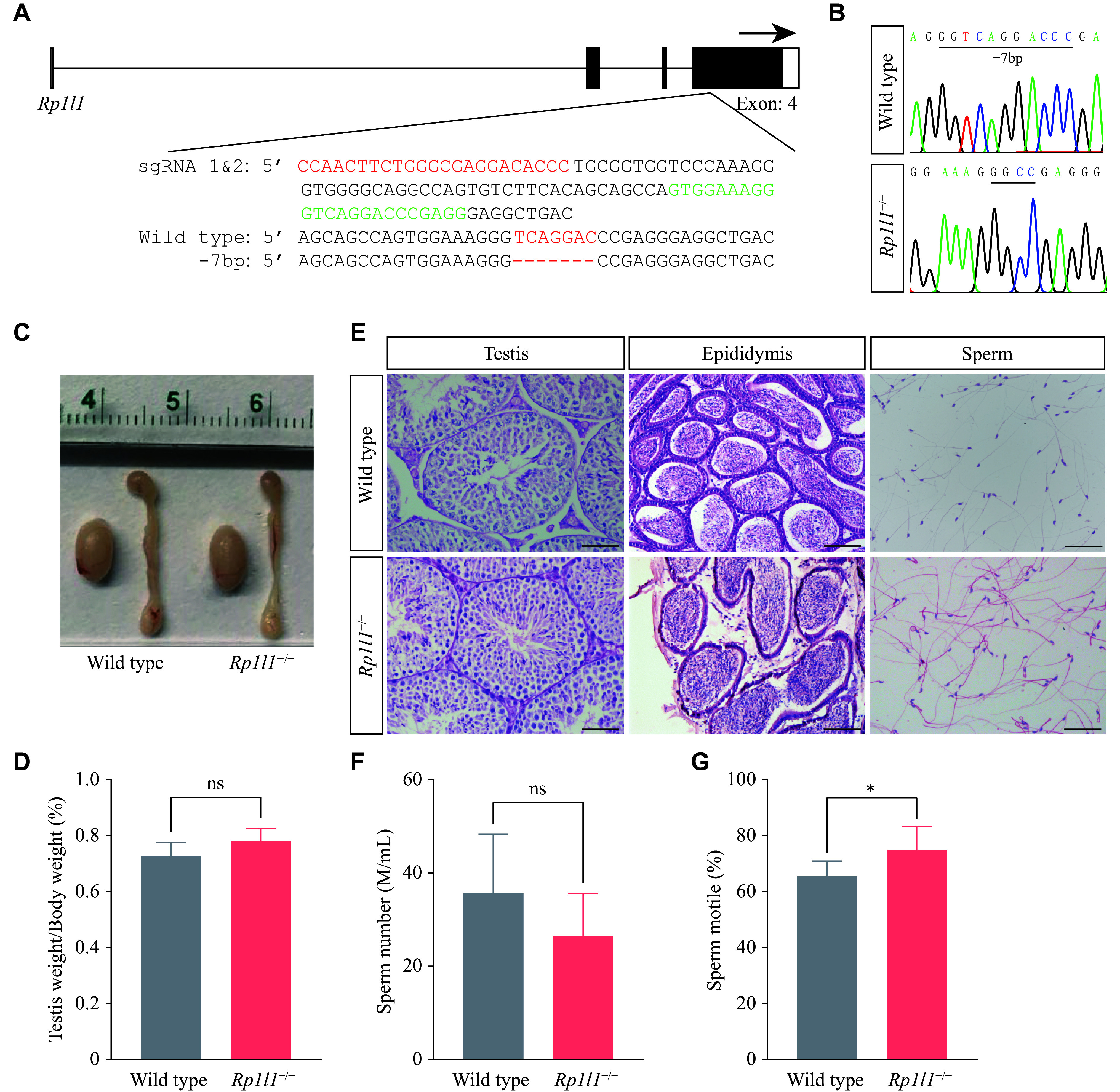
Phenotypic analysis of *Rp1l1*^−/−^ male mice. A: Gene editing strategy for *Rp1l1*. Two sgRNAs were designed to target exon 4 of *Rp1l1*. B: Genotyping of *Rp1l1*^−/−^ mice through PCR and subsequent Sanger sequencing. Lines indicate the mutant alleles. C: Morphology of the testis from wild-type and *Rp1l1*^−/−^ mice. A cm-scale ruler is shown above for size reference. D: Comparison of bilateral testis weight to body weight between wild-type mice and *Rp1l1*^−/−^ mice. *n* = 3 males per group. E: Histological analysis of the testes, epididymides, and sperm in wild-type and *Rp1l1*^−/−^ mice. Testis sections were stained with PAS; epididymal sections and sperm smears were stained with HE. Scale bars, 50 μm (testes), 200 μm (epididymides), and 50 μm (sperm). F: Comparison of sperm concentration between wild-type and *Rp1l1*^−/−^ mice. *n* = 3 males per group. M/mL, million per milliliter. G: Comparison of sperm motility between wild-type and *Rp1l1*^−/−^ mice. *n* = 3 males per group. Quantitative data of D, F, and G are presented as mean ± standard deviation (SD), and statistical analysis was performed using two-tailed unpaired Student's *t*-test. Abbreviation: ns, not significant.

*Rp1l1* was mutated using two sgRNAs targeting exon 4 (***Fig. 5A***). Primers for detecting the mutant alleles are presented in ***Supplementary Table 2***. The resulting mutant allele, carrying a 7-bp deletion in exon 4, was confirmed by PCR and Sanger sequencing (***Fig. 5B***). We then collected and analyzed the morphological and histological features of the testes and epididymides. We found no significant differences in testis appearance (***Fig. 5C***), testis weight (***Fig. 5D***), and testis histology (***Fig. 5E***). The results of CASA also showed that homozygous adult *Rp1l1*^−/−^ male mice displayed normal sperm concentration (***Fig. 5F***) and even had a higher proportion of motile spermatozoa than wild-type mice (***Fig. 5G***).

**Figure 6:**
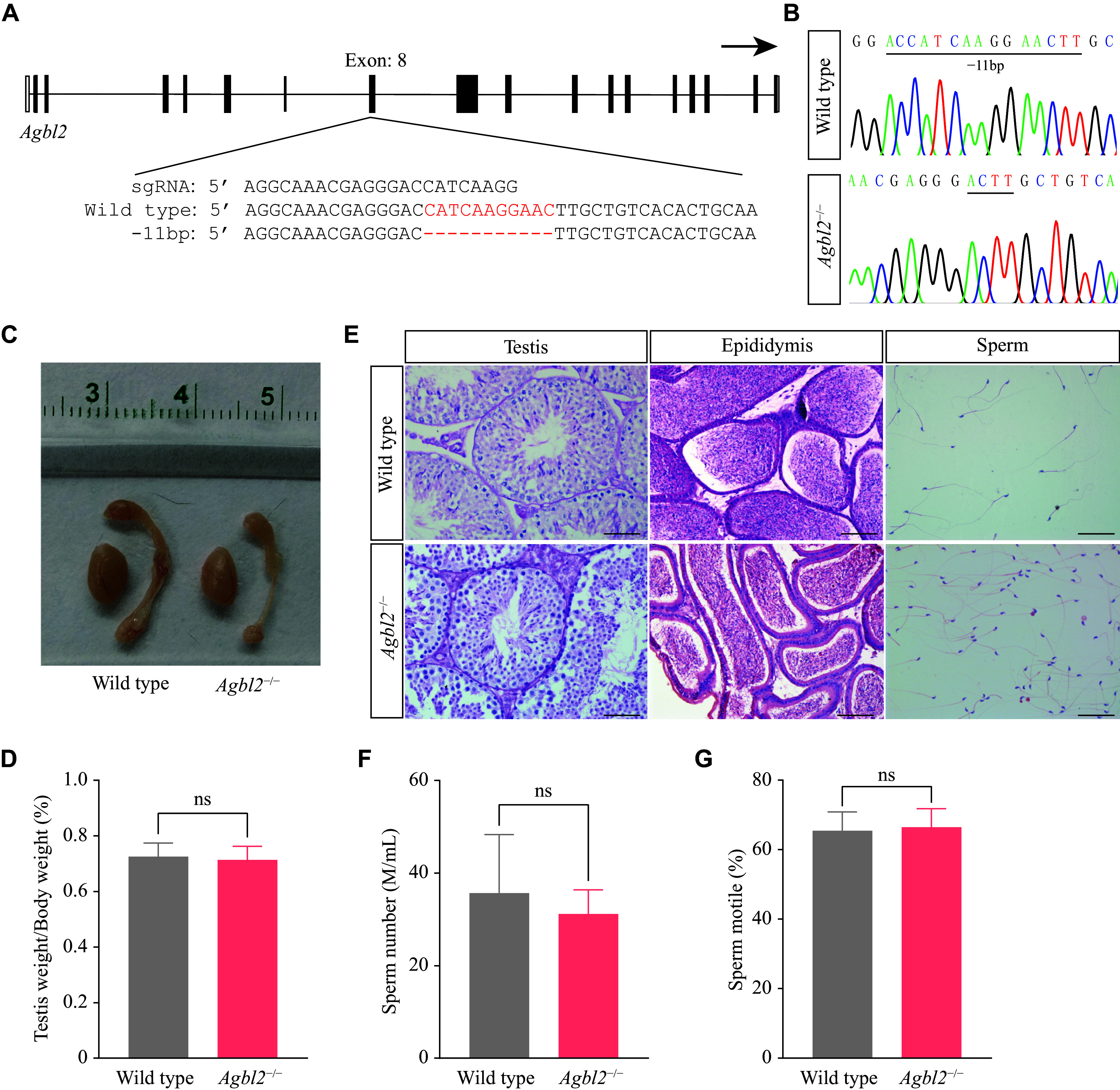
Phenotypic analysis of *Agbl2*^−/−^ male mice. A: Gene editing strategy for *Agbl2*. An sgRNA was designed to target exon 8 of *Agbl2*. B: Genotyping of *Agbl2*^−/−^ mice through PCR and subsequent Sanger sequencing. Lines indicate the mutant alleles. C: Morphology of the testes from wild-type and *Agbl2*^−/−^ mice. A cm-scale ruler is shown above for size reference. D: Comparison of bilateral testis weight to body weight between wild-type mice and *Agbl2*^−/−^ mice. *n* = 3 males per group. E: Histological analysis of the testes, epididymides, and sperm in wild-type and *Agbl2*^−/−^ mice. Testis sections were stained with PAS; epididymal sections and sperm smears were stained with HE. Scale bars, 50 μm (testes), 100 μm (epididymides), and 50 μm (sperm). F: Comparison of sperm concentration between wild-type and *Agbl2*^−/−^ mice. *n* = 3 males per group. M/mL, million per milliliter. G: Comparison of sperm motility between wild-type and *Agbl2*^−/−^ mice. *n* = 3 males per group. Quantitative data of D, F, and G are presented as mean ± standard deviation (SD), and statistical analysis was performed using two-tailed unpaired Student's *t*-test. Abbreviation: ns, not significant.

One sgRNA was used to target exon 8 of *Agbl2*, resulting in an 11-bp deletion in exon 8 (***Fig. 6A***). The genotypes were confirmed by PCR using the primers shown in ***Supplementary Table 2***. Sanger sequencing revealed that the mutant mouse had an 11-bp deletion (***Fig. 6B***). Homozygous *Agbl2*^−/−^ mice were viable. No significant differences were found in the morphology of the testis (***Fig. 6C***) and the testis-to-body weight ratio (***Fig. 6D***). Histological analyses of the testes and epididymides from *Agbl2*^−/−^ male mice revealed no abnormalities or defects in any stage of spermatogenesis and spermatozoa morphology compared with control testis and epididymis sections (***Fig. 6E***). The CASA results of *Agbl2*^−/−^ mice showed no statistically significant differences in sperm concentration (***Fig. 6F***) and sperm motility (***Fig. 6G***).

**Figure 7:**
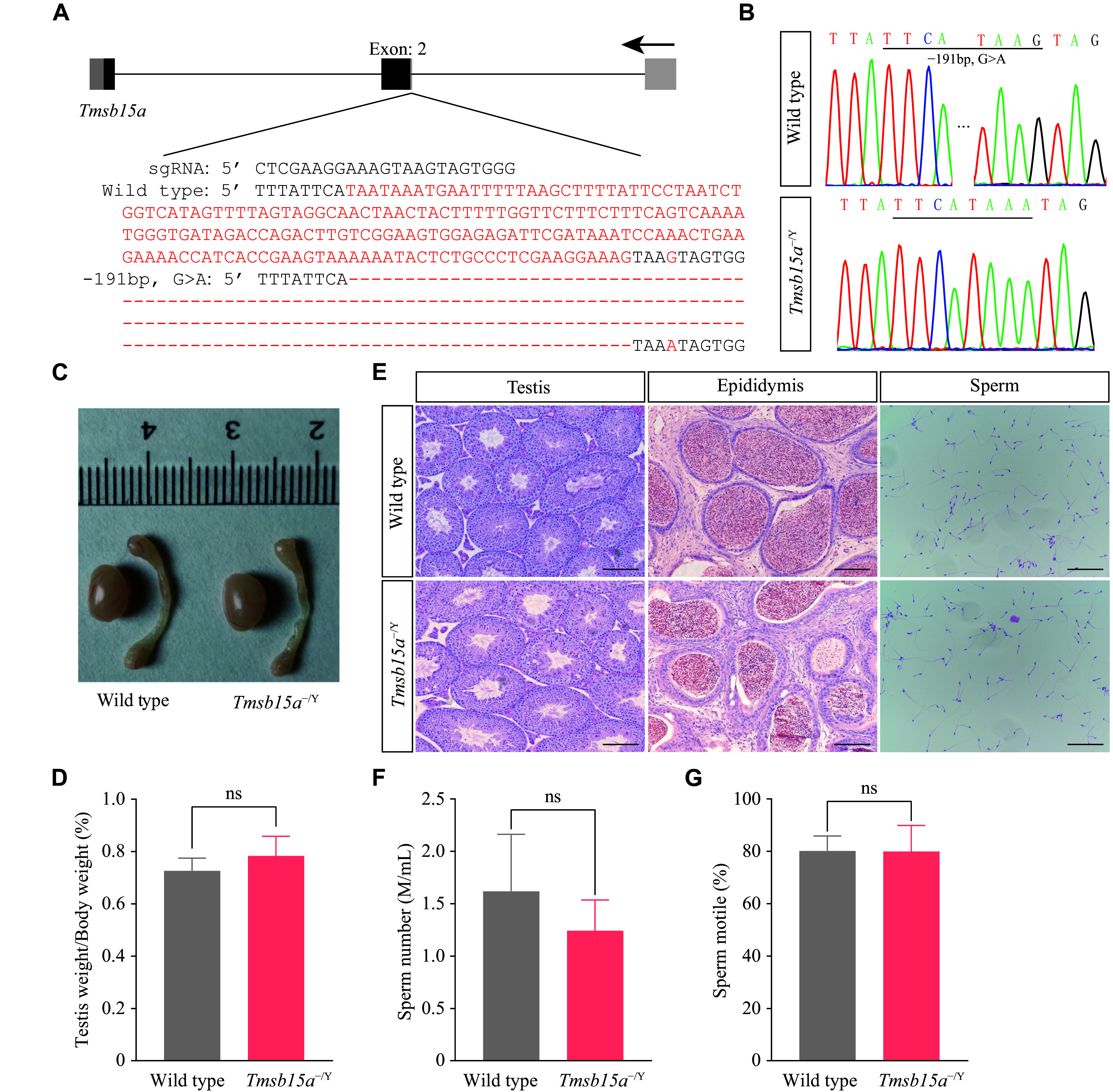
Phenotypic analysis of *Tmsb15a*^−/Y^ male mice. A: Gene editing strategy for *Tmsb15a*. An sgRNA was designed to target exon 2 of *Tmsb15a*. B: Genotyping of *Tmsb15a*^−/Y^ mice through PCR and subsequent Sanger sequencing. Lines indicate the mutant alleles. C: Morphology of the testes from wild-type and *Tmsb15a*^−/Y^ mice. A cm-scale ruler is shown above for size reference. D: Comparison of bilateral testis weight to body weight between wild-type mice and *Tmsb15a*^−/Y^ mice. *n* = 3 males per group. E: Histological analysis of the testes, epididymides, and sperm in wild-type and *Tmsb15a*^−/Y^ mice. Testis sections were stained with PAS; epididymal sections and sperm smears were stained with HE. Scale bars, 100 μm (testes), 100 μm (epididymides), and 100 μm (sperm). F: Comparison of sperm concentration between wild-type and *Tmsb15a*^−/Y^ mice. *n* = 3 males per group. M/mL, million per milliliter. G: Comparison of sperm motility between wild-type and *Tmsb15a*^−/Y^ mice. *n* = 3 males per group. Quantitative data of D, F, and G are presented as mean ± standard deviation (SD), and statistical analysis was performed using two-tailed unpaired Student's *t*-test. Abbreviation: ns, not significant.

*Tmsb15a*^−/Y^ mice were generated using one sgRNA designed to disrupt exon 2 (***Fig. 7A***), resulting in a 191-bp deletion and a 1-bp mutation, which led to the complete loss of exon 2. Genotyping was validated by PCR and Sanger sequencing (***Fig. 7B***). The primers used for identifying the mutant alleles are summarized in ***Supplementary Table 2***. As in all previously mentioned mutant mice, no obvious changes in the morphology of *Tmsb15a*^−/Y^ testes were observed (***Fig. 7C***), and no significant differences were found in the testis weight/body weight compared with wild-type mice (***Fig. 7D***). Histological analyses of *Tmsb15a*^−/Y^ testes and epididymides showed that all stages of spermatogenesis and the morphology of spermatozoa were normal (***Fig. 7E***). Additionally, CASA results confirmed that sperm concentration and the percentage of motile spermatozoa did not differ significantly between *Tmsb15a*^−/Y^ and control mice (***Fig. 7F*** and ***7G***).

## Discussion

Male infertility is a complex condition influenced by multiple factors, with genetic disorders identified as one of the primary contributors^[2]^. Our genome-wide association studies (GWAS) have identified numerous common variants associated with male infertility^[26–29]^. Additionally, rare variants in multiple genes, the functional impairment of which may result in male infertility, have also been reported^[30–33]^. Nevertheless, the pathogenic mechanisms underlying the condition in many affected individuals remain poorly understood^[34]^. Therefore, screening the function of genes that play essential roles in male reproduction using mouse models can significantly enhance our understanding of the etiology of male infertility^[35–36]^. In the current study, we generated six mutant mouse lines using the CRISPR/Cas9 system and demonstrated that these genes are not individually essential for male reproduction, suggesting that these genes may also not play critical roles in male fertility.

Among the six mutant mouse lines, we observed that *Tekt3* deficiency significantly reduced sperm motility, although mutant males remained fertile. This phenomenon may be attributed to several compensatory mechanisms. First, the maintenance of sperm count and mating behavior may compensate for the impaired motility. Despite reduced sperm motility, normal sperm counts likely ensure a sufficient number of gametes to sustain fertility. A previous study reported that double knockout of *Tekt3* and *Tekt4* led to reduced average litter size without altering total monthly offspring^[37]^, suggesting that mating behavior may play a compensatory role. Second, functional redundancy among Tektin family members may contribute. Cao *et al*^[38]^ demonstrated that *Tekt3* co-localizes with *Tekt1*, *Tekt2*, *Tekt4*, and *Tekt5* in the peri-axonemal accessory structures of the flagellum. Therefore, we hypothesize that other Tektins may partially compensate for Tekt3 deficiency, preserving normal fertility in mice.

The current study primarily investigates the phenotypic effects of individual disruption of six genes, without exploring potential synergistic interactions. Given possible redundant or cooperative functions, combined knockouts may reveal abnormal reproductive phenotypes. Future studies could also explore synergistic regulatory mechanisms among these genes to further elucidate the molecular basis of spermatogenesis and male fertility.

Several traditional strategies have been used to identify candidate pathogenic genes associated with male infertility. These include: (1) conducting whole-exome or whole-genome sequencing in patients to detect pathogenic variants, followed by prioritizing candidate genes based on conservation scores and expression patterns, with subsequent functional validation in mouse models^[39]^; and (2) investigating genes with unknown functions or of particular interest within known biological pathways related to spermatogenesis, such as piRNA pathways and RNA-binding proteins^[40–41]^, followed by reproductive phenotype analysis in mouse models. However, candidate pathogenic genes identified by these approaches often do not consistently impair male fertility in mouse models as anticipated. Therefore, new strategies are needed.

We propose that supervised machine learning could be applied to identify essential genes for male reproduction. A study used an ensemble machine learning approach to predict meiosis-essential proteins based on their distinct abundance patterns, raising the enrichment of meiosis-essential proteins from 6.54% to 49.30%, with four of 10 mutant mice showing meiotic arrest in validation^[42]^. Although predictive accuracy remained imperfect, finding a new approach to more accurately predict the function of a given gene could substantially enhance our understanding of the functions of previously uncharacterized genes.

Human-mouse heterogeneity presents an unavoidable challenge in studying pathogenic genes associated with male infertility, contributing significantly to the discrepancy where genes identified as risk factors in humans often fail to exhibit abnormal reproductive phenotypes in mouse models. Although the vast majority of mouse protein-coding genes have human orthologs^[9]^, the genetic basis of human male infertility is highly heterogeneous, involving complex multigenic interactions and environmental factors^[43]^. In contrast, mouse models often rely on single-allele knockouts or mutations, which may fail to fully recapitulate this complexity. For example, we did not detect abnormal reproductive phenotypes in these six mutant mouse models, while non-obstructive azoospermia patients do carry rare predicted loss-of-function variants in these genes (***Supplementary Table 7***). It is important to note that the pathogenicity of these variants is determined by bioinformatic analyses; whether these variants are diagnostic pathogenic variants or not requires further evaluation of population allele frequencies, inheritance patterns, and familial cosegregation. Another potential explanation is the unique epigenetic regulation and transcriptional dynamics involved in human spermatogenesis, which may not be fully replicated by the expression patterns or functions of specific key genes in mice^[44]^. Integrating human and mouse transcriptomic and proteomic data into the training of machine learning models may help bridge the gap caused by the heterogeneity between humans and mice, thereby enhancing the translatability of findings from mouse models to humans. It is essential to recognize the limitations inherent in the present study. The *Mlf1*^R243*/R243*^ mouse line, which targets a conserved sequence identified in patients with TOF, constitutes a hypomorphic allele retaining partial protein function rather than a complete null mutation. Consequently, our results do not definitively establish that *Mlf1* is non-essential for male fertility, as residual protein activity may mitigate the effects of the mutation. Similarly, although *Rp1l1* is relatively highly expressed in the testis, its low detection in scRNA-seq datasets, likely attributable to technical dropout or infrequent expression, necessitates cautious interpretation. Notably, despite this discrepancy, males harboring *Rp1l1* mutations exhibited a significantly greater proportion of motile spermatozoa compared to controls. While the biological significance of this increased motility remains to be fully elucidated, these observations underscore that individual gene disruption can lead to distinct, albeit non-sterilizing, alterations in sperm parameters. Potential CRISPR-Cas9 off-target mutations were also not experimentally evaluated in this study, meaning unlinked secondary mutations cannot be entirely ruled out. Moreover, our phenotypic screening is subject to statistical limitations. The modest sample size (*n* = 3–4) restricts the capacity to detect subtle defects (*e.g.*, age-dependent subfertility or competitive mating impairments). Therefore, our findings primarily demonstrate the absence of overt fertility deficits rather than absolute equivalence. Additionally, while all lines were maintained on a consistent C57BL/6 background, the employment of age-matched colony controls rather than littermate controls may introduce potential background genetic variability, potentially affecting the sensitivity of our screening. Future studies with larger cohorts, littermate controls, and complete knockout strategies will be necessary to definitively validate these findings.

In summary, we found that disruption of these six genes did not cause overt male infertility under standard laboratory breeding conditions. The current study focused solely on reproductive phenotypes, without evaluating abnormalities in other systems. Therefore, all lines will be made available as bioresources on reasonable request for further analysis by other researchers. Publishing these results on non-essential genes can help other research communities save time and avoid redundant efforts. Consequently, when variants in these genes are identified by whole-exome or whole-genome sequencing, the present findings may provide useful supporting evidence for variant prioritization; however, potentially pathogenic variants should not be excluded solely on the basis of these mouse phenotypes, particularly given possible species- and allele-specific effects.

## Additional information

The online version contains supplementary material available at http://www.jbr-pub.org.cn/article/doi/10.7555/JBR.39.20250247. The data and materials used in this article will be shared on reasonable request to the lead contact, Zhibin Hu (zhibin_hu@njmu.edu.cn).
