# Supplementary material for: CRISPR/Cas9-mediated genome editing identifies six testis-expressed genes individually dispensable for overt male fecundity in mice
Source: J Biomed Res. 2026 Jul 28;40(4):410–24. doi: 10.7555/JBR.39.20250247 (PMC13438311; doi:10.7555/JBR.39.20250247)
Supplement: Supplementary file 1 [file jbr-40-4-410-S1.pdf]

Available online at www.jbr-pub.org.cn

Open Access at PubMed Central

Journal of Biomedical Research, 2026 40(4): 1–6

Supplementary Data

JBR

CRISPR/Cas9-mediated genome editing identifies six testis-expressed genes individually dispensable for overt male fecundity in mice

Heling Fu[1,△], Haoran Chen[1,2,△], Chenmeijie Li[1], Shuai Lu[1], Yayun Gu[1], Zhibin Hu[1,2,✉]

[1]*State Key Laboratory of Reproductive Medicine and Offspring Health, Nanjing Medical University, Nanjing, Jiangsu 211166, China;*
[2]*Department of Epidemiology, Center for Global Health, School of Public Health, Nanjing Medical University, Nanjing, Jiangsu 211166, China.*

Supplementary Table 1 **Single-guide RNA sequences used for CRISPR/Cas9 to generate knockout or knock-in mice**

Gene symbol	sgRNA sequence (5′–3′)	Mutation
Efcab7	AAATGACGGTTACTCTTTACAGG	G>C
Tekt3	GAGTCAATGACTTAGCGTTTTGG<br>GACTGATATTAAGAGAAGGCTGG	−76bp
Mlf1	TCATGAGCATCCAGGGTCACGGG	CGG>TGA
Rpl1l	GGGTGTCCTCGCCCAGAAGTTGG<br>GTGGAAAGGGTCAGGACCCGAGG	−7bp
Agbl2	AGGCAAACGAGGGACCATCAAGG	−11bp
Tmsb15a	CTCGAAGGAAAGTAAGTAGTGGG	−191bp, G>A

△These authors contributed equally to this work.

✉Corresponding author: Zhibin Hu, E-mail: zhibin_hu@njmu.edu.cn, ORCID: 0000-0002-8277-5234.

Received: 19 June 2025; Revised: 25 October 2025; Accepted: 26 October 2025; Available online: 28 October 2025; Published date: 28 July 2026

CLC number: R394.3; R698.2, Document code: A

The authors reported no conflict of interests. Zhibin Hu is the Editor-in-Chief of this journal and was excluded from all editorial decisions. The manuscript was handled independently, with peer review conducted by at least two external reviewers.

This is an open access article under the Creative Commons Attribution (CC BY 4.0) license, which permits others to distribute, remix, adapt and build upon this work, for commercial use, provided the original work is properly cited.

© 2026 by Journal of Biomedical Research.

https://doi.org/10.7555/JBR.39.20250247

Supplementary Table 2 **PCR primers for Sanger sequencing**

Gene symbol	Primer (5′–3′)	Annealing temperature
Efcab7-F	CCATTAAAGCCTGGTCAGTATGA	57 ℃
Efcab7-R	GTTCACAAACTCCATTGGTTCCC	
Tekt3-F	AATTCCTCACGGCACAACTCAGA	57 ℃
Tekt3-R	GCTTCTATGGGCAGCAGAAATGT	
Mlf1-F	AGTAACCAGGTAGTGAATACGA	57 ℃
Mlf1-R	ATGCTACTATGTCAGGCTACTT	
Rp1l1-F	CTTGGTGGCTGCTGATGAT	57 ℃
Rp1l1-R	CTGTCTCCAGTGGCTCTTG	
Agbl2-F	GCATTCTGAGTGGAGCATTAGC	57 ℃
Agbl2-R	TGCCAGCCCTTAATAAGTTCTCT	
Tmsb15a-F	GACTTGTCGGAAGTGGAGAGAT	57 ℃
Tmsb15a-R	AGAGCAAACCTTGAGTCTGGAA	

Supplementary Table 3 **PCR primers for detecting expression in different mouse tissues**

Gene symbol	Primer (5′–3′)	Annealing temperature
Efcab7-exp-F	CTGGGAGTCCTCGAACAAAG	60 ℃
Efcab7-exp-R	TGGTCTTTTGGGATGGATTT	
Tekt3-exp-F	TGCAGGAGATCTTCCAGATTG	60 ℃
Tekt3-exp-R	GGCTTTCACAGCCAAGTCAT	
Mlf1-exp-F	GATCACGATGTTCCGGATGC	60 ℃
Mlf1-exp-R	TTCGCCATCATCACGTTCTC	
Rp1l1-exp-F	CTCTGAGCTTCTGCGCTTTC	60 ℃
Rp1l1-exp-R	ACTTTGTTTGGCGTTTGGTC	
Agbl2-exp-F	GGTGATTCCCATGTTAAACCCA	60 ℃
Agbl2-exp-R	CTTCCCGTTCCTTCTTTGCA	
Tmsb15a-exp-F	TGGCCCTGAAGCTAACTGAA	60 ℃
Tmsb15a-exp-R	CTTGCCAGTGTTGGCATTTA	

Supplementary Table 4 **Detailed information of average litter size per male mouse across six mouse lines**

Genotypes	Male ID	Litter sizes	No. of recorded litters	Total pups (*n*)	Mean litter size
WT	WT-1	10, 6, 9	3	25	8.33
WT	WT-2	9, 10, 8	3	27	9.00
WT	WT-3	6, 9, 9	3	24	8.00
WT	WT-4	10, 9, 10	3	29	9.67
Efcab7	Efcab7-183#	6, 8, 9, 7	4	30	7.50
Efcab7	Efcab7-179#	8, 11, 7, 9	4	35	8.75
Efcab7	Efcab7-290#	6, 11, 9, 7	4	33	8.25
Tekt3	Tekt3-74#	8, 7, 9	3	24	8.00
Tekt3	Tekt3-53#	9, 8, 7	3	24	8.00
Tekt3	Tekt3-54#	9, 9	2	18	9.00
Mlf1	Mlf1-124#	8, 9, 10, 8, 8	5	43	8.60
Mlf1	Mlf1-74#	10, 11, 11	3	32	10.67
Mlf1	Mlf1-89#	8, 11, 10, 11	4	40	10.00
Mlf1	Mlf1-91#	8, 9, 10, 8	4	35	8.75
Rp1l1	Rp1l1-260#	9, 10, 8, 6	4	33	8.25
Rp1l1	Rp1l1-319#	8, 6, 7, 9	4	30	7.50
Rp1l1	Rp1l1-450#	8, 10, 7, 8, 9, 9	6	51	8.50
Agbl2	Agbl2-74#	7, 8, 8, 9	4	32	8.00
Agbl2	Agbl2-62#	8, 9, 6, 8	4	31	7.75
Agbl2	Agbl2-65#	9, 8, 9, 7	4	33	8.25
Tmsb15a	Tm-N2-60	8, 7, 7, 9	4	31	7.75
Tmsb15a	Tm-N2-69	9, 10, 8, 10	4	37	9.25
Tmsb15a	Tm-N2-47	7, 8, 8, 7	4	30	7.50

Supplementary Table 5 **Variant information detected in *EFCAB7*, *TEKT3*, *MLF1*, *RP1L1*, and *AGBL2* in whole-exome sequencing data from tetralogy of Fallot cases**

Gene	Variant	Impact
EFCAB7	ENST00000371088:c.683-1G>C:NA	Splice site
TEKT3	ENST00000338696:c.546_547delGCinsT:p.Glu182fs	Frameshift
	ENST00000338696:c.544G>T:p.Glu182*	Nonsense
MLF1	ENST00000392822:c.778C>T:p.Arg260*	Nonsense
RP1L1	ENST00000382483:c.4663C>T:p.Gln1555*	Nonsense
	ENST00000382483:c.2651delC:p.Pro884fs	Frameshift
	ENST00000382483:c.1972C>T:p.Arg658*	Nonsense
	ENST00000382483:c.676A>T:p.Arg226*	Nonsense
AGBL2	ENST00000357610:c.2683_2684delTC:p.Ser895fs	Frameshift
	ENST00000357610:c.742C>T:p.Arg248*	Nonsense

Supplementary Table 6 **The information on the proportion of sperm abnormalities in wild-type and mutant mice**

Gene symbol	Genotype	Calculated sperm numbers	Tapered	Banana-shaped	Macrocephalic	Immature	Double-headed	Double-tailed	Tail coiled	Total teratozoospermia rate
Wild type	+/+	369	0.81%	0.54%	0.00%	3.25%	0.27%	0.54%	3.25%	8.67%
Efcab7	c.509-1G>C/c.509-1G>C	726	0.00%	0.41%	0.14%	1.52%	0.69%	0.00%	9.50%	12.26%
Tekt3	−76bp/−76bp	841	0.00%	0.24%	0.00%	1.31%	0.36%	0.12%	9.99%	12.01%
Mlf1	R243*/R243*	775	0.00%	0.00%	0.13%	1.55%	0.26%	0.00%	12.00%	13.94%
Rp1l1	−7bp/−7bp	748	0.00%	0.00%	0.00%	1.74%	0.27%	0.00%	11.63%	13.64%
Agbl2	−11bp/−11bp	524	0.19%	0.76%	1.15%	0.76%	0.38%	0.00%	11.83%	15.08%

Supplementary Table 7 **Pathogenic loss-of-function variant information in NOA whole-genome sequencing data**

Gene	Variant	Impact
EFCAB7	ENST00000371088:c.769C>T:p.Arg257*	Nonsense
TEKT3	ENST00000338696:c.734+1delG:NA	Splice site
	ENST00000338696:c.604C>T:p.Arg202*	Nonsense
	ENST00000338696:c.544G>T:p.Glu182*	Nonsense
	ENST00000338696:c.483_486delTCAT:p.Ile161fs	Frameshift
	ENST00000338696:c.340C>T:p.Arg114*	Nonsense
	ENST00000338696:c.229G>T:p.Glu77*	Nonsense
MLF1	ENST00000392822:c.696_697delTG:p.Ser232fs	Frameshift
	ENST00000392822:c.778C>T:p.Arg260*	Nonsense
RP1L1	ENST00000382483:c.6418G>T:p.Glu2140*	Nonsense
	ENST00000382483:c.5405_5414delAAACTGGGGG:p.Glu1802fs	Frameshift
	ENST00000382483:c.4936C>T:p.Gln1646*	Nonsense
	ENST00000382483:c.4624C>T:p.Arg1542*	Nonsense
	ENST00000382483:c.3424A>T:p.Lys1142*	Nonsense
	ENST00000382483:c.1972C>T:p.Arg658*	Nonsense
	ENST00000382483:c.121C>T:p.Arg41*	Nonsense
AGBL2	ENST00000357610:c.695-1G>A:NA	Splice site

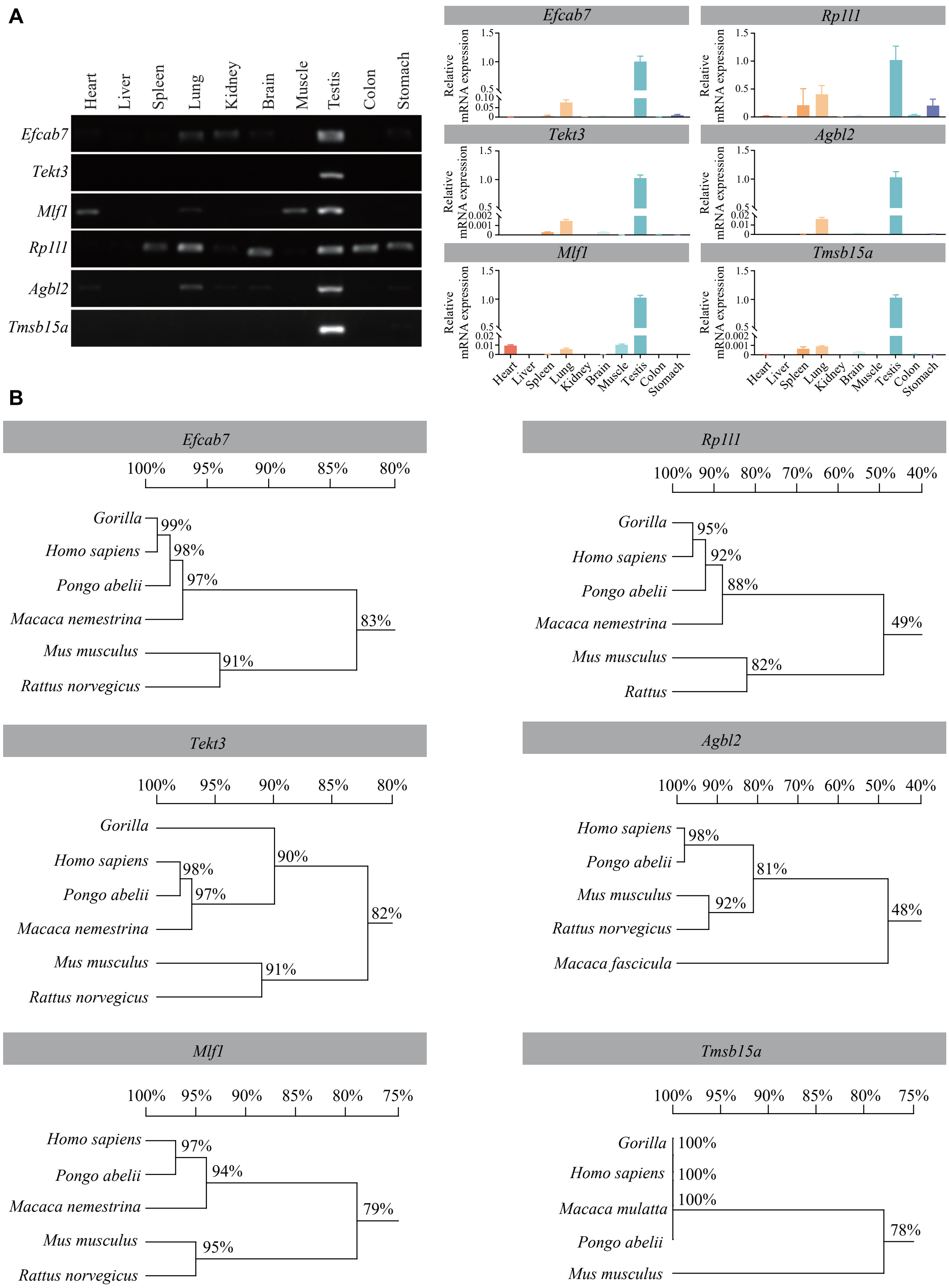

Supplementary Fig. 1 **Expression patterns and phylogenetic trees of six genes in the mouse.** A: Expression of the six genes in different mouse tissues. Conventional reverse transcription PCR (RT-PCR) was performed and validated by gel electrophoresis. *Gapdh* was used as a loading control to normalize sample loading across different tissues. Quantitative real-time RT-PCR (qRT-PCR) was also performed to confirm the expression of these six genes. Data are presented as mean ± standard deviation (n = 3). B: Phylogenetic trees for all six genes were constructed using DNAMAN software.

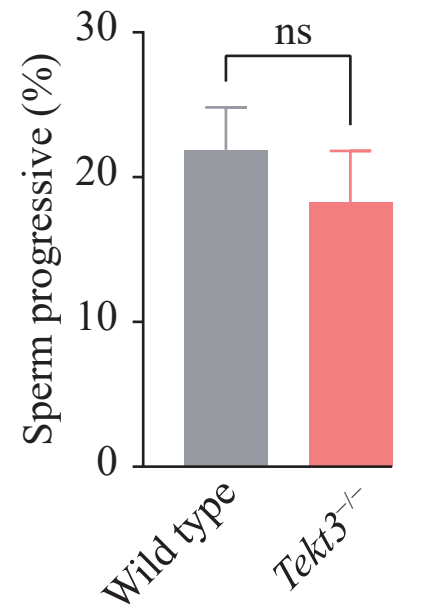

Supplementary Fig. 2 **Comparison of sperm progressive motility between wild-type and *Tekt3*$^{-/-}$ mice.** Quantitative data are presented as mean ± SD (n = 3 males per group). Statistical analysis was performed using a two-tailed unpaired Student's t-test. Abbreviation: ns, not significant.

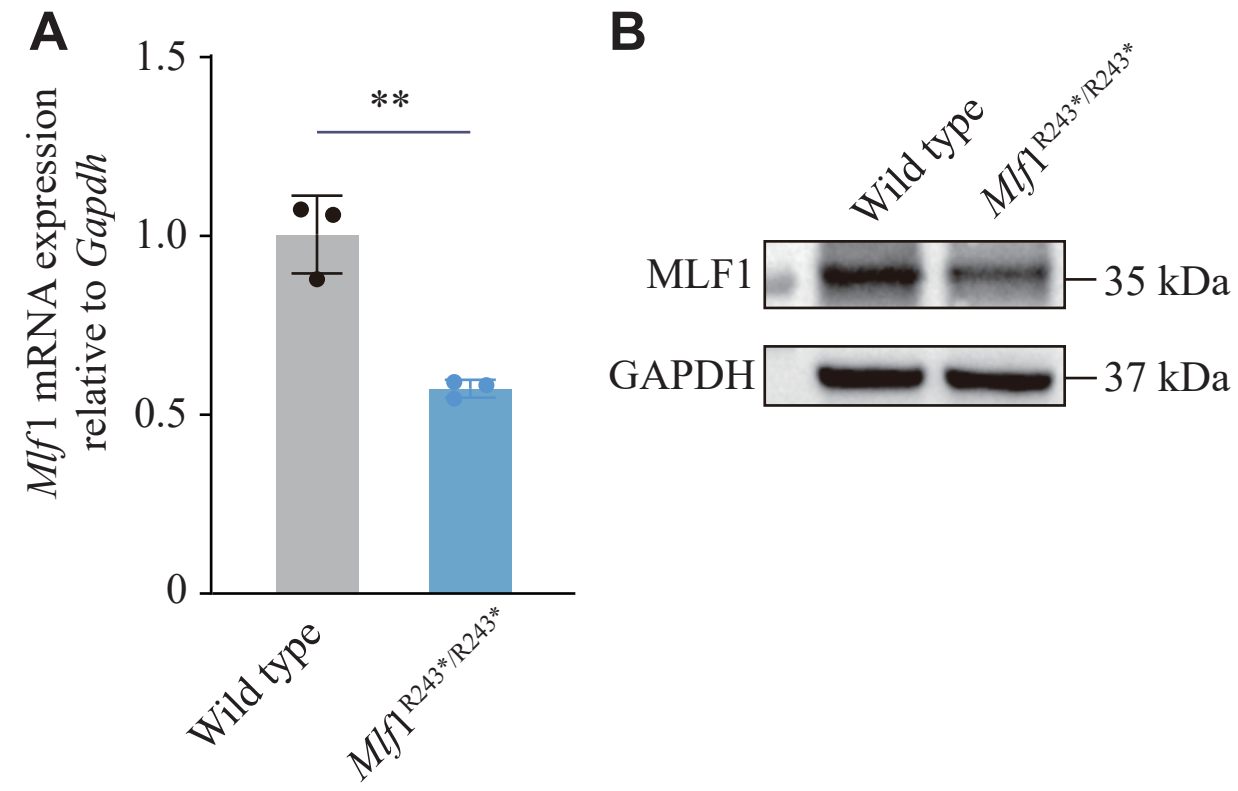

Supplementary Fig. 3 **Expression of *Mlf1* mRNA and protein in wild-type and *Mlf1*$^{R243*/R243*}$ mice.** A: Relative *Mlf1* mRNA expression normalized to *Gapdh*. Data are presented as mean ± standard deviation (n = 3). Statistical significance was determined by an unpaired two-tailed Student's t-test. $^{**}P$ < 0.01. B: MLF1 protein expression was analyzed by Western blotting, with GAPDH used as a loading control.
